# Oxidative Stress in Hypoxic-Ischemic Encephalopathy: Molecular Mechanisms and Therapeutic Strategies

**DOI:** 10.3390/ijms17122078

**Published:** 2016-12-10

**Authors:** Mingyi Zhao, Ping Zhu, Masayuki Fujino, Jian Zhuang, Huiming Guo, IdrisAhmed Sheikh, Lingling Zhao, Xiao-Kang Li

**Affiliations:** 1 Department of Pediatrics, the Third Xiangya Hospital, Central South University, Changsha 410006, China; zhaomingyi1983@aliyun.com (M.Z.); Dr_idris99@outlook.com (I.S.); 2 Guangdong Cardiovascular Institute, Guangdong General Hospital, Guangdong Academy of Medical Sciences, Guangzhou 510100, China; zhupingzggd@126.com (P.Z.); zhuangjianzggd@aliyun.cn (J.Z.); guohuiming@vip.tom.cn (H.G.); 3 National Research Institute for Child Health and Development, 2-10-1 Okura, Setagaya-ku, Tokyo 157-8535, Japan; nekonagashi@hotmail.com; 4 National Institute of Infectious Diseases, 1-23-1 Toyama, Shinjuku-ku, Tokyo 162-8640, Japan

**Keywords:** oxidative stress, hypoxic-ischemic encephalopathy, cell damage, therapeutic strategy

## Abstract

Hypoxic-ischemic encephalopathy (HIE) is one of the leading causes of morbidity and mortality in neonates. Because of high concentrations of sensitive immature cells, metal-catalyzed free radicals, non-saturated fatty acids, and low concentrations of antioxidant enzymes, the brain requires high levels of oxygen supply and is, thus, extremely sensitive to hypoxia. Strong evidence indicates that oxidative stress plays an important role in pathogenesis and progression. Following hypoxia and ischemia, reactive oxygen species (ROS) production rapidly increases and overwhelms antioxidant defenses. A large excess of ROS will directly modify or degenerate cellular macromolecules, such as membranes, proteins, lipids, and DNA, and lead to a cascading inflammatory response, and protease secretion. These derivatives are involved in a complex interplay of multiple pathways (e.g., inflammation, apoptosis, autophagy, and necrosis) which finally lead to brain injury. In this review, we highlight the molecular mechanism for oxidative stress in HIE, summarize current research on therapeutic strategies utilized in combating oxidative stress, and try to explore novel potential clinical approaches.

## 1. Introduction

Hypoxic-ischemic encephalopathy (HIE), which is induced by the disruption of cerebral blood flow and a subsequent lack of oxygen to the affected area, is one of the leading causes of increased risk of death and lifelong disability, such as visual impairment, learning impairment, epilepsy, mental retardation, blindness, and cerebral palsy [1]. The prevention and treatment of this disease remains a severe medical issue with global financial repercussions. One of the most widely accepted pathophysiological mechanisms of HIE involves the generation of oxidative stress.

Because of high concentrations of sensitive immature cells, metal-catalyzed free radicals, non-saturated fatty acids, and low concentrations of antioxidant enzymes, the brain requires high levels of oxygen supply and is, thus, extremely sensitive to hypoxia [2]. Following the hypoxic-ischemic event, oxidative stress, which later triggers the release of oxygen and nitrogen species, calcium overloading, free radical generation, excitotoxicity, acidotoxicity, ionic imbalance, inflammation, apoptosis, autophagy, and necrosis, plays an important role in pathogenesis [3].

Strong evidence indicates that when the delicate balance between pro-oxidants and antioxidants tips toward a more oxidative state, local reactive oxygen species (ROS) production is dramatically increased [4]. If available in appropriate amounts, ROS have been shown to act as signal transduction molecules providing cell protection and maintain in homeostasis. In contrast, when exceeding the buffering capacity of scavenging molecules and antioxidant enzymes, ROS are shown to cause significant damage to biological macromolecules, such as proteins (membrane protein degeneration), lipids (lipid oxidation), and nucleic acids (DNA degeneration) [5,6]. These derivatives induce a complex interplay of multiple pathways including immune defense, cell signaling, and induction of mitogenesis. In this review, we highlight the molecular mechanism for oxidative stress in HIE and examine potentially powerful clinical therapeutic strategies.

## 2. Overview for ROS Formation, Decomposition, and Sources

The main formation and decomposition of ROS has been shown previously [6]. In human tissues, sources of ROS production include the NADPH oxidases (NOX), xanthine oxidize (XO), arachidonic acid metabolism pathways (12/15 lipoxygenase), uncoupled nitric oxide synthase (NOS), and the mitochondrial electron transport system. A large body of evidence suggests that NOX and NOS, along with an oxygen-starved mitochondrial electron transport system, comprise the major sources of ROS in the brain during hypoxia and ischemia [7,8,9,10,11,12,13,14,15,16].

After a hypoxic-ischemic attack, resident immune cells in the brain are stimulated. They generate several oxygen free radicals and then induce the expression of pro-inflammatory mediators [17]. These superoxide (including superoxide anions (O_2_·^−^), hydrogen peroxide (H_2_O_2_), hypochlorous acid and hydroxyl radicals (·OH) O_2_·^−^) are generated via several enzyme systems, including NADPH oxidase, glutathione peroxidase (GPX), cyclooxygenase (COX), xanthine dehydrogenase, XO, monoamine oxidase (MAO), and myeloperoxidase (MPO). However, the role of NADPH oxidase, the most important source of ROS, in neonatal inflammatory responses following hypoxic-ischemic attack is controversial. On the one hand, inhibition of NADPH oxidase increases the level of inflammation cytokines [18], however, by comparison, in vivo studies have showed that it can exacerbate inflammatory responses and worsen neurological outcomes in animal models [18,19].

## 3. Clinical Manifestations and Potential Biomarkers for Hypoxic-Ischemic Encephalopathy (HIE)

During and after exposure to HIE, neural imaging, electroencephalography (EEG), and biochemical markers have been used to assess prognosis and predict long-term outcomes, such as visual impairment, learning impairment, epilepsy, mental retardation, blindness, cerebral palsy, and even death. As the spectrum of imaging findings relates to the evolution of ischemic parenchymal tissue (basal ganglia and thalami, corticospinal tract, white matter, and cortex), magnetic resonance image (MRI) is the preferred imaging choice. Doppler sonography is also sensitive in the detection of HIE. Because of the high water content in the brain, and high protein content of the cerebrospinal fluid, which results in poor parenchyma contrast resolution, computed tomography (CT) is the least sensitive modality for the evaluation of HIE [20,21,22,23]. Like imaging techniques, EEG can be readily measured at the bedside. The most promising EEG features in identifying HIE include burst suppression, low voltage, and a flat trace [24]. Various numbers of biochemical markers in body fluids have also been suggested to be useful as sentinel biomarkers, including S100B, neuron-specific enolase (NSE), miRNA, lactate dehydrogenase (LDH), adrenomedullin, activin A, Tau protein, non-protein bound iron, serum CD4 cell count, nuclear factor-κB (NF-κB), ionized calcium, creatine kinase (CK-BB), carboxyl-terminal esterase L1 (UCH-L1), glial fibrillary acidic protein (GFAP), and interleukins, IL-6, IL-8, and IL-1β [20,25,26,27].

## 4. Pathogenesis and Molecular Mechanisms

### 4.1. Inflammation-Mediated Oxidative Stress in HIE

Both clinical and experimental studies suggest that oxidative stress plays a role in crosstalk between inflammatory systems and creates “windows of susceptibility” to HIE. Two phases of HIE-induced oxidative stress mediated by inflammatory responses have been identified. In the initial step, inflammation is activated by amoeboid microglia, the resident immune cell in the brain. Microglia respond vigorously to hypoxic-ischemic attack and produce excess inflammatory cytokines (IL-1β, TNF-α, etc.) along with glutamate, nitric oxide (NO), and ROS [28,29]. Alongside microglia, astrocytes are also activated by ROS, and secrete pro-inflammatory cytokines (IL-6, TNF-α, IL-1α, and IL-1β) and IFN-γ. Rapid increases in the levels of these cytokines cause collective accumulation in the brain tissue, leading to direct injury through increasing levels of toxic NO, inducing the apoptosis of neuronal cells, inhibiting neurogenesis, and attracting immune cells to the ischemic site [30]. Brain tissue injury is exacerbated by the complex interaction between neutrophils, lymphocytes, adhesion molecules, cytokines, and chemokines. During ischemia, neutrophils can amplify brain injury through inducing ROS production. Interestingly, however, studies have shown that lymphocytes play a negative role in the pathogenesis of the acute ischemic brain [31,32,33]. The main cytokines relating to the inflammatory responses seen in HIE are IL-1, IL-6, IL-10, TNF-α, and TGF-β, with high levels of these cytokines under oxidative stress correlating positively with HIE severity [34,35,36,37,38].

### 4.2. Mitochondrial Injury in HIE

Mitochondria function as “power houses”, generating adenosine triphosphate (ATP), and serve as the major sites of oxidative metabolism [39,40]. During HIE, oxidative stress caused by ROS “bursts” plays an important role in changes in the mitochondria, including ischemic starvation, reperfusion-induced hyper activation, mitochondrial dysfunction, and delayed neuronal death [41]. Initially, during oxidation associated with mitochondrial respiration, the mitochondrial membrane potential (ΔΨm) becomes positive in the inner chamber, in contrast to physiological conditions. Secondly, there is an increase of intracellular Ca^2+^, with decreasing glucose and ATP generation, and cytochrome c (CytC) releases into the cytoplasm. During HIE, the release of CytC from the mitochondrial serves as a rather important pathway for the cascades of apoptotic events. First, loosely-coupled or tightly-bound CytC is damaged in the mitochondrial membrane and then released; second, pro-apoptotic proteins, Bid, Bad, Bax, Bak, Bok, and Bim in the outer mitochondrial membrane increase the permeability of the membrane, forming specific pores and stimulating free CytC release; third, CytC binds to apoptosis protein-associated factor 1 (Apaf-1) and forms the Apaf-1/caspase-9/CytC complex. Finally, caspase-3 is activated, which triggers apoptosis and delayed neuronal death [42,43].

### 4.3. Oxidative Stress Mediated Apoptosis in HIE

Apoptosis, the process of programmed cell death, is a series of well-coordinated and strictly controlled processes leading to cell blebbing, shrinkage, nuclear fragmentation, chromatin condensation, proteolysis, and chromosomal DNA fragmentation [44]. Apoptosis occurs in a wide variety of physiological and pathological situations [45,46]. In recent years, ROS have become a target for drug discovery since their production is characteristic of the early stages of apoptosis preceding the collapse of the mitochondrial membrane potential, release of pro-apoptotic factors, and activation of caspases [47]. Oxidative stress-mediated apoptosis in HIE can occur via extrinsic and intrinsic pathways. In extracellular apoptotic signaling, inflammatory markers (such as TNF-α, TRAIL, Apo3L, and Fas ligand (Fas-L)) respond to HIE, activate NF-κB, bind to their receptors, lead to caspase-8 activation and the cleavage of the pro-apoptotic Bcl-2 family protein Bid to t-Bid, and finally induce both apoptosis and cell survival triggering intrinsic signals. Oxidative stress-mediated mitochondrial injury appears to be mainly involved in the intracellular apoptotic signaling. The first mechanism is mediated by intermembrane space proteins, such as CytC, apoptosis inducing factor (AIF), Endo G and Smac/DIABLO (second mitochondria-derived activator of caspase/direct IAP binding protein with a low pI), released into the cytosol. The second mechanism is mediated by proteins of the Bcl-2 family acting directly on the outer mitochondrial membrane.

### 4.4. Oxidative Stress Mediated Autophagy in HIE

Autophagy, or type-II programmed cell death, is a conserved, intracellular, lysosome-dependent degradation process that recycles defective proteins or damaged organelles. Autophagy is up-regulated in response to oxidative stress, helping to restore intracellular homeostasis by disposing a number of harmful molecules such as misfolded proteins overflowing from endoplasmic reticulum (ER) stress, cytosolic proteins damaged by ROS, or even dysfunctional mitochondria or ER from prolonged oxidative stress [48,49,50,51]. The autophagy pathway can induce IKKα/NF-κB/I-κB kinase β-mediated pro-inflammatory signaling via the oxidative stress pathway [52,53]. In parallel, when homeostasis of ROS is disrupted, excessive ROS are accumulated in the mitochondria and cytoplasm and can cause oxidative damage to cells and induce autophagy [54]. ROS-mediated molecular networks, such as PI3K-Akt-mTOR, TLR-4, IGF, MAPK, and AMPK, depend on several distinct mechanisms involving catalase or caspase activation of autophagy-related genes, and disturbances in the mitochondrial electron transport chain [55]. However, oxidative stress-mediated autophagy in HIE remains controversial, and whether there is a beneficial or detrimental role of autophagy depends on whether intracellular stresses have been resolved.

## 5. Clinical Strategies of Antioxidants in HIE

**Figure 1:**
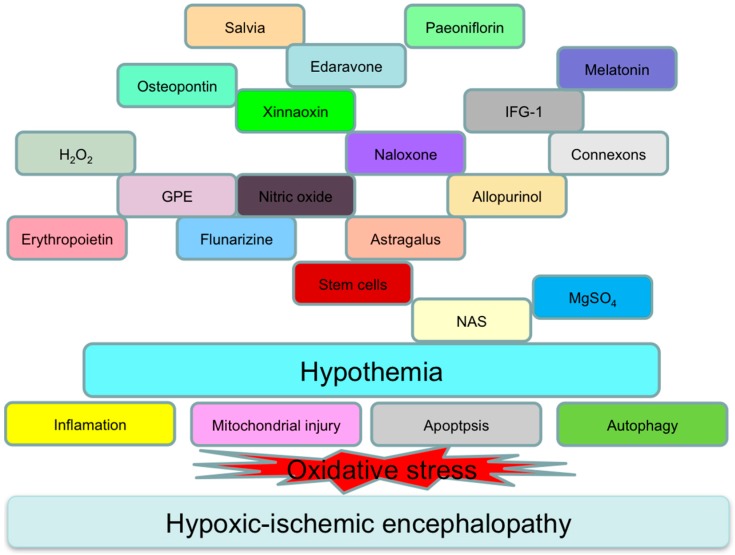
Interventional targets following oxidative stress in hypoxic-ischemic encephalopathy (HIE). NAS, *N*-acetylserotonin; GPE, the N-terminal tripeptide of Insulin-like growth factor-1 (IGF-1).

Over the past decade, hypothermia has been established as the standard treatment for HIE, and further investigation for a multi-targeted approach has yet to be researched in depth. Interventional targets have consisted of pathways involved in inflammation, apoptosis, and autophagy followed by oxidative stress in experimental translational studies (Figure 1).

### 5.1. Hypothermia

Hypothermia is routinely used as a protective therapeutic tool for moderate to severe HIE in clinical application [56]. Selective head cooling (34.5 °C) and total body cooling (33.5 °C) are the two therapeutic hypothermia methods. The decrease in temperature affects all physiological systems of the body, including redistribution of blood flow and disturbances of microcirculation, eventually leading to the reduction of metabolism and oxygen supply to tissues. When hypothermia is used in HIE it maintains or improves the level of antioxidants [57]. Therapeutic hypothermia must begin within the first six hours after birth, and be maintained for three days. Studies have shown that this treatment is effective for reducing cerebral injury and improves the brain outcome secondary to hypoxic-ischemic attack in full-term-born and near-term preterm newborns [58].

### 5.2. Erythropoietin (EPO)

There is compelling preclinical research and clinical evidence that(EPO) can promote the expression of anti-apoptotic genes relative to pro-apoptotic genes, inhibit inflammation, attenuate oxygen free radicals, decrease caspase activation, and increase neurogenesis in response to HIE through the cross talk between PI3K/AKT, STAT5, and ERK molecular signal pathway [59,60,61]. Currently there are two active clinical trials (Neonatal Erythropoietin and Therapeutic Hypothermia Outcomes in Newborn Brain Injury (NCT01913340) and Efficacy of Erythropoietin to Improve Survival and Neurological Outcome in Hypoxic Ischemic Encephalopathy (NCT01732146)) examining EPO in combination with hypothermia in infants with HIE. The NCT01913340 assesses an EPO dose of 1000 U/kg/dose IV × 5 doses, while the NCT01732146 evaluates EPO intravenous injections (5000 U/0.3 mL) 1000 to 1500 U/kg/dose three times given every 24 h with the first dose within 12 h of delivery [62,63]. Studies have shown that EPO administration is safe in both the adult and neonatal brain. Combining cooling with EPO in HIE could improve the recovery of sensor motor function, behavioral and cognitive performance, and histological integrity, improve motor and cognitive responses, promote cerebella growth, and reduce death or disability [64,65,66].

### 5.3. N-Acetyl-5-methoxytryptamine (Melatonin)

Melatonin, a strong endogenous indolamine, has shown a neuroprotective role. Melatonin, has anti-inflammatory, antioxidant, and anti-apoptotic properties in HIE. Melatonin′s protective actions are believed to stem from the interaction of its receptors (receptor dependent actions), its direct free radical scavenging (receptor-independent actions), and because of yet-undefined functions. Melatonin freely crosses the placenta and blood-brain barrier making it more attractived [67,68,69]. A previous study has shown that pretreated melatonin in asphyxiated term neonates (received hypothermia combined application with melatonin 10 mg/kg × 5 days, oral.) is feasible and may ameliorate brain injury [70]. It is likely that higher doses are required to obtain an antioxidant effect and this high dose might even desensitize the melatonin receptors.

### 5.4. N-Acetylserotonin (NAS)

NAS has also been shown to be better as scavenging peroxyl radicals than melatonin itself. The neuroprotective effects of NAS in HIE may result through its effects on cells mitochondrial impairment, including permeability transition pore opening, fragmentation, inhibition of the subsequent release of apoptogenic factors from mitochondria into the cytoplasm, activation of apoptosis protein expression, as well as suppression of the activation of autophagy under oxidative stress conditions [71].

### 5.5. Magnesium Sulfate (MgSO_4_)

A lot of interesting research shows that MgSO_4_ can reduce secondary inflammation and associated injury that occurs under oxidative stress. The possible mechanism may be mainly due to the fact that MgSO_4_ can bind to the magnesium site on *N*-methyl-d-aspartate (NMDA) glutamate channels, inhibiting free radical production, and stabilizing the cell membrane [72,73,74,75]. There is now evidence from meta-analyses of randomized controlled trials that antenatal administration of MgSO_4_ is associated with a small but significant reduction in the risk of cerebral palsy and gross motor dysfunction after preterm birth [76,77]. However, it has no significant effect on death or disability.

### 5.6. Stem Cells

Combined treatment with hypothermia and transplantation stem cells (e.g., amnion epithelial cells (AECs), hematopoietic stem cells (HSC), umbilical cord blood (UCB), UCB-derived endothelial progenitor cells (EPCs), and bone marrow-derived mesenchymal stem cells (MSCs)) has been regarded as a therapeutic strategy to promote functional recovery in animal models of HIE. A previous study has shown that the collection, preparation, and infusion of fresh autologous UCB cells for use in infants with HIE is feasible [78]. In addition to stem cell transplantation, there is ongoing research in the field of stem cell factors (e.g., G-CSF) [79,80,81,82].

### 5.7. Edaravone (3-Methyl-1-phenyl-2-pyrazolin-5-one)

Edaravone is a free radical scavenger that is thought to be useful in the treatment of post-ischemic neuronal dysfunction, and improving memory and learning ability in HIE. Using brain microdialysis, electron paramagnetic resonance (EPR) spectroscopy, photo acoustic imaging, and laser speckle contrast imaging detection, research on edaravone has indicated that it is believed to interact with peroxyl and hydroxyl radicals, lipid peroxidation, and DNA peroxidation, and that it creates a radical intermediate that forms stable oxidation products. Clinical studies show that, in the treatment group treated with edaravone alone or in combination which other neuroprotective drugs (such as Ganglioside, hyperbaric oxygen (HBO), Xingnaojing, etc.) the difference of national institutes of health stroke scale (NIHSS), proinflammatory, anti-inflammatory cytokines, free radicals was statistically significant [83,84,85,86].

### 5.8. Allopurinol

Evidence exists that suggest that allopurinol, a xanthine-oxidase inhibitor, functions as a chelator of non-protein bound iron, as well as a direct scavenger of free radicals, suggesting that allopurinol may be an adjunct to therapeutic hypothermia in HIE [87,88,89,90]. In a recent study, treatment with allopurinol in HIE infants with hypoplastic left heart syndrome significantly impeded the biochemical cascade of neuronal damage, including seizures, death, coma or cardiac events, in comparison to a control group [91].

### 5.9. Osteopontin (OPN)

Recent studies show that OPN, a multifunctional glycoprotein, is up-regulated in brain tissue affected by HIE. Exogenous OPN has decreased infarct volume and improved neurological outcomes. OPN-induced neuroprotection was associated with cleaved caspase-3 inhibition, regulation of cerebral cell proliferation, oligodendrocyte differentiation, and anti-apoptotic cell death [92,93,94].

### 5.10. Flunarizine

Flunarizine, a selective Ca^2+^ channel blocker, which has potent neuroprotective properties against hypoxic-ischemic encephalopathy, acts according to a mechanism independent of effects on dopamine release [95].

### 5.11. Nitric Oxide

Inducible NO synthase (iNOS) is induced to produce excessive NO in which leads to cascade reactions of inflammation and neuronal death in HIE. Nitric oxide, an iNOS inhibitor, via the NF-κB/nNOS pathway plays a neuroprotective role by increasing iron deposition, inhibiting platelet and leukocyte adhesion, maintaining cerebral blood flow, and preventing neuronal injury. NO has been shown to be a new therapeutic agent in the treatment of brain hypoxia-ischemia [11,12,96,97].

### 5.12. Hydrogen Peroxide (H_2_O_2_)

Most reviewed studies demonstrate that H_2_O_2_, either in the gas or liquid form, acts as a treatment for hypoxia/ischemia through preconditioning protection. Assessed by 2,3,5-triphenyltetrazoliumchloride (TTC), Nissl and TUNEL staining, and caspase-3 and caspase-12 activities in the cortex and hippocampus, H_2_O_2_ may act via theHIF-1α pathway inhibiting neuronal apoptosis and attenuating cerebrovascular reactivity (CR) to hypercapnia, *N*-methyl-d-aspartate (NMDA), norepinephrine, and sodium nitroprusside [98,99,100,101].

### 5.13. Insulin-Like Growth Factor-1 (IGF-1) and the N-Terminal Tripeptide of IGF-1 (GPE)

IGF-1 and GPE is a polypeptide hormone that has been investigated as a potential neurotrophic factor for the treatment of HIE. IGF-1, via the PI3K/Akt/GSK3β and NF-κB pathway phosphorylation, attenuates activation of caspases and mitogenic effects. Previous research has demonstrated that the neuroprotective actions of IGF-1 infusion were global, robust, and displayed a broad effective dose range and treatment window [102].

### 5.14. Blockade of Connexin Hemi Channels (Connexons)

Increasing evidence supports that suppressing the induction or activity of the connexin proteins forming hemichannels contributes to HIE. Previous research has revealed that unopposed connexons also play an important role. They mediate the release of paracrine molecules, which in turn transmit cell death messages by the secretion of intracellular mediators (such as ATP, Nicotinamide adenine dinucleotide (NAD^+^), and glutamate), which ultimately leads to cell edema [103,104].

### 5.15. Naloxone and β-FNA

Naloxone, a µ-opioid receptor blocker, and β-FNA, a µ-opioid receptor antagonist, both attenuate myeloperoxidase activity and chemokine (macrophage inflammatory protein-1 alpha and -2) mRNA expression via C-fos, C-jun, Nur77, and the MAPK pathway in HIE [105].

### 5.16. Salvia

Studies have shown that Endothelin-1 (ET-1), NO, and CK-BBin in both the blood and cerebrospinal fluid (CSF) participated in the pathological process and determine the therapeutic effects of HIE. *Salvia* injection demonstrated a therapeutic effect superior to a control group at a statistically significant level [106].

### 5.17. Other Traditional Chinese Medicines and Related Extracts

This group includes examples such as paeoniflorin, xinnaoxin, astragalus, and safflower, among others.

## 6. Conclusions

HIE is one of the leading causes of morbidity and mortality in neonates. In this review, we highlighted the molecular mechanism and protection strategies for oxidative stress in HIE, mainly focusing on mechanisms related to anti-inflammation, anti-apoptosis, and regulation of autophagy (Table 1). Therapeutic interventions enabling the prevention or reduction in hypoxia-induced brain damage before or during an early stage of free radical production will require continued investigation to determine optimal effectiveness. Moreover, the safety and efficacy of these combinatorial strategies for HIE can be maximized by following pertinent translational research guidelines.

## Figures and Tables

**Table 1:** Clinical strategies of antioxidants in HIE and their proposed molecular mechanisms.

Clinical Strategies of Antioxidants	Proposed Molecular Mechanisms Related to Neuroprotection	Reference
Hypothermia	reduces cerebral injury and improves the brain outcome secondary to HI attack	[56,58]
Erythropoietin (EPO)	anti-apoptotic, inhibits inflammation, attenuates oxygen free radicals, decreases caspase activation, and increases neurogenesis	[59,60,61,62,63,64,65,66]
Melatonin	anti-inflammation, antioxidant, and anti-apoptotic [66,67,68,69]	[67,68,69,70]
*N*-acetylserotonin (NAS)	impairs mitochondrial, activates apoptosis, and suppress autophagy	[71]
Magnesium Sulfate (MgSO_4_)	binds to the magnesium site on NMDA glutamate channels, inhibits free radical production, and stabilizes the cell membrane	[73,74,75,76,77]
Stem cells	increases neurogenesis and angiogenesis	[78,79,80,81,82]
Edaravone	creates a radical intermediate that forms stable oxidation products	[83,84,85,86]
Allopurinol	a chelator of non-protein bound iron and scavenger of free radicals	[87,88,89,90,91]
Osteopontin (OPN)	cleaved caspase-3 inhibition, regulates cerebral cell proliferation, oligodendrocytes differentiation, and is anti-apoptotic	[92,93,94]
Flunarizine	dopamine release	[95]
iNOS	increases iron deposition, inhibits platelet and leukocyte adhesion, and maintains cerebral blood flow	[96,97]
Hydrogen peroxide (H_2_O_2_)	inhibits neuronal apoptosis and attenuates cerebrovascular reactivity	[98,99,100,101]
IGF-1 & GPE	attenuates activation of caspases and mitogenic effects	[102]
connexons	mediates the release of paracrine molecules	[103,104]
Naloxone and β-FNA	attenuates myeloperoxidase activity and chemokine mRNA expression	[105]
Traditional Chinese medicines and related extracts	such as *Salvia*, paeoniflorin, xinnaoxin, astragalus, safflower, and so on	[106]

## Abbreviations

EEG	Electroencephalography
HIE	Hypoxic-ischemic encephalopathy
ROS	Reactive oxygen species
SOD	Superoxide dismutase
